# Child-Driven Assessment of Plate Waste and Food-Waste Awareness in Primary Schools

**DOI:** 10.3390/foods15122231

**Published:** 2026-06-20

**Authors:** Barbara Peraboni, Vanessa Lupetti, Vera Lavelli

**Affiliations:** Department of Food, Environmental and Nutritional Sciences (DeFENS), University of Milan, 20133 Milan, Italy; peraboni.barbara@gmail.com (B.P.); vanessa.lupetti@gmail.com (V.L.)

**Keywords:** plate waste, school canteen, primary school, awareness, food waste education, child-driven assessment

## Abstract

Food waste in school canteens is widely recognized as a significant issue because of its economic consequences, environmental impact, and implications for children’s health. Previous studies have used robust methods to quantify this problem and assess mitigation strategies. This case study of primary school children (6–11 years) used a child-driven approach to measure plate waste and explore reasons for uneaten food and concern about waste. The results indicated that a group of volunteer children (n = 104) directly involved in the assessment were able to evaluate their peers’ food waste, obtaining estimates comparable to those reported in previous studies (mean: 108.4 g per child). The students for whom food waste was measured (n = 443) took part in interviews and proved to be active participants capable of evaluating their own context, although their level of engagement could be further strengthened. Among children who reported leaving food uneaten, a substantial proportion provided specific reasons; nevertheless, generic explanations accounted for 26% of responses for the first course and 35% for the second. Approximately 78.5% of the children demonstrated a high level of sensitivity to food waste, recognizing its direct effects (wasting their parents’ money), indirect effects (waste in a broader sense), and social effects (world hunger/poverty). Establishing a baseline for children’s sensitivity to their own food waste is therefore needed, as it could serve as an indicator of both the urgency and the effectiveness of educational interventions.

## 1. Introduction

Food waste generated within school canteens represents a relevant operational and economic inefficiency and leads to unnecessary environmental burdens [1,2,3] as well as avoidable losses in the nutritional value of meals provided to students [4,5,6,7,8,9]. The substantial environmental impact associated with daily catering activities was evidenced by a comparative analysis of life-cycle-based emission factors across 21 public services, which indicated that school canteens constitute one of the principal sources of energy consumption and greenhouse-gas emissions within the public sector [1]. This outcome is attributable to multiple stages of the food supply chain, including raw material procurement, food production and distribution, meal consumption, the use of disposable tableware, and the management of food and material waste [1]. In European countries, carbon emissions per meal served at school was estimated to be 0.84–1 kg CO_2_ equivalent for Croatia, 0.95–1.05 kg CO_2_ equivalent for Italy, 1.03–1.35 kg CO_2_ equivalent for Serbia, 1.20–1.27 kg CO_2_ equivalent for UK, 1.87–2.41 kg CO_2_ equivalent for Greece [2] and 0.83 kg CO_2_ equivalent in Sweden [3]. Consequently, preventing the accumulation of food waste represents a key strategy for reducing the environmental impact of school canteens.

The effect of food waste in school canteens on the nutritional adequacy of children’s diets is another important issue, particularly at the elementary school level. In fact, unhealthy dietary patterns can lead to health problems during childhood and in later life, since eating habits developed early tend to shape long-term dietary behavior [4]. Accordingly, the age range of 5–11 years is often identified as the optimal period for implementing strategies to prevent common diseases such as obesity [5,6]. A large-scale survey conducted in Portugal estimated that, each day, approximately 211 kcal, 10.3 g of fat, 15.8 g of carbohydrates, 13.1 g of protein, and 1.6 g of fiber were discarded per child [7]. Changes in the geographical and cultural context in which a primary school is located can lead to substantial differences in plate-waste levels. For example, the daily caloric loss from plate waste was 125 kcal per elementary pupil in Thailand [8]. In another study performed in Sweden, the estimated loss per meal was lower, i.e., 23.9 kcal, 0.6 g of fat, 4.6 g of carbohydrates and 1.5 g per protein [9]. Although the available data cannot be generalized to broader populations or different institutional settings, the relevance of food waste in educational food services is undeniable.

Within this context, the systematic monitoring and reduction in food waste in school canteens constitute a key intervention area, with children aged 6–11 years representing a particularly strategic target group for maximizing community-level impact [10]. The accumulation of food waste in educational settings has been extensively documented, with several surveys highlighting its persistently high levels [8,11,12,13,14]. One of the most recent approaches consists of measuring food waste using artificial intelligence-based procedures which may provide abundant and less observer-dependent data [11]. Most previous studies performed on children at the primary school observed children’s attitudes and behaviors toward food waste from an “external” perspective, aiming to design managerial strategies to encourage healthier food choices [8,12,13] or to decrease the environmental and cost impact of the canteen meal [14].

A few studies have directly involved children in self-assessment activities, adopting a child-driven approach to reducing food waste. One small-scale study engaged 25 children in a primary school, who received several educational sessions and were involved in different activities aimed at raising awareness of food waste. Food waste was measured prior to and after these actions and around a 30% food-waste reduction at lunch was observed in the intervention group but not in the other children [15]. In a large-scale study, educational interventions such as providing feedback to diners through plate-waste trackers, implementing pedagogic meals, and organizing kitchen workshops were tested to lower plate waste in school canteens across Europe (Austria, Germany, and Sweden). The plate-waste tracker alone led to a 17% reduction in plate waste, while other interventions were not effective [16].

The current study adopts a child-driven approach to the analysis of food waste, with the objective of determining: (a) the potential for children to be actively engaged in food-waste monitoring; (b) the general awareness among children of the quantity of food they discard; and (c) the degree and nature of their sensitivity toward the impacts of food waste.

## 2. Materials and Methods

### 2.1. Schools

This study was conducted at two public primary schools (totaling 547 children aged 6–11 years) with similar socio-economic characteristics and size, both belonging to the same municipality and located in an urban area in Northern Italy. The schools shared the same canteen committee and catering company. School 1 had 251 pupils in 12 classes: two first-grade classes (n = 34, aged 6–7), two second-grade classes (n = 39, aged 7–8), two third-grade classes (n = 43, aged 8–9), two fourth-grade classes (n = 51, aged 9–10), and four fifth-grade classes (n = 84, aged 10–11). School 2 had 296 pupils in 14 classes: two first-grade classes (n = 46, aged 6–7), three second-grade classes (n = 59, aged 7–8), three third-grade classes (n = 57, aged 8–9), three fourth-grade classes (n = 70, aged 9–10), and three fifth-grade classes (n = 64, aged 10–11).

### 2.2. Menu

The daily menu employed in this study consisted of rice (first course), cheese (second course), and broccoli (side dish). This specific combination was intentionally selected to maximize participation by ensuring that the meal was accessible to all students. In particular, foods containing gluten were excluded to accommodate children with celiac disease, while meat and fish were omitted to respect students who avoid these products for religious, environmental, and/or health-related reasons and follow dietary practices such as vegetarian or vegan diets.

### 2.3. Study Design

**Figure 1:**
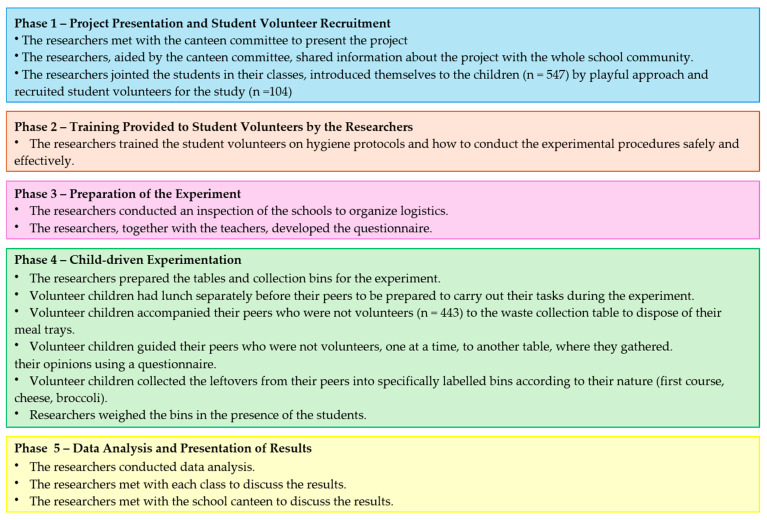
Flowchart of the methodological approach.

The flowchart of the methodological approach used to involve children in food-waste collection and peer-to-peer interviews is shown in Figure 1.

In the first phase, the researchers presented the project to the canteen committee, which was composed of representatives of the parents, delegates from the catering company that provides the meals, members of the canteen staff, and the municipal official responsible for the area where the schools are located. The canteen committee played a relevant role in involving the whole school community, including teachers and families. Parents were informed about the procedures and were asked to sign an informed consent form when they agreed on participation. Then, the researchers joined the students in their classrooms and introduced the project using a pedagogically informed, playful approach. For pupils in the first and second grades, illustrated materials depicting food waste and correct or inappropriate environmental behaviors (disposing of food waste on the ground or in the bin) were provided. After a brief instructional explanation, children were asked to color the correct actions, aiming to foster early awareness through age-appropriate visual activities. Students in the third, fourth, and fifth grades were divided into two groups per class and completed a non-competitive quiz game developed by the researchers to assess their ability to identify appropriate actions related to common food waste (Table 1). The quiz answers were provided orally and collectively and were not included in the analysis. During these sessions, four student volunteers from each class (n = 104) were recruited to actively participate in the food-waste assessment and assist the researchers; in case more volunteers than needed offered their contribution, they were enrolled in chronological order. The remaining students (n = 443) were invited to complete the questionnaire, ensuring the participation of all children in the study.

The second phase was a ‘training’ phase. The pupils who decided to take part in the analysis as volunteers and helpers were trained to work safely and to carry out the activities effectively. The training was conducted by dividing the pupils into age groups—1st and 2nd grade, and 3rd, 4th, and 5th grade—so as to interact with them using age-appropriate language. The importance of not contaminating the plates was explained, including hygiene notions such as the awareness of allergens. The rules were also explained: wearing gloves, tying up hair or wearing a cap, and not drinking or eating during the experiment.

The third phase involved an inspection of the schools by the researchers during the lunch break to identify the most suitable area for placing the tables for collecting the trays at the end of the meal, the bins for gathering the food waste produced during lunch, and the tables needed to administer the questionnaires to the students. The questionnaire was developed by the researchers together with the teachers (one per class, n = 26) to ensure its clarity. Compared with the first draft, only one question was removed, namely, “*Do you more often finish everything or leave something uneaten*?”, as it was considered ambiguous. The final questionnaire consisted of short questions without response options, so as not to influence the children (Table 2).

Answers to Questions 1 and 2 were classified as “Yes” or “No.” In the case of affirmative responses, a single reason was requested and based on children’s responses, motivations were grouped into three categories: those related to sensory dislike, those related to feelings of satiety, and those reflecting generic reasons. Answers to Questions 3, 4, and 6 were classified as “Yes,” “No,” or “Not sure.” Answers to Question 5 were classified as “Yes” or “No.” In the case of affirmative responses, a single motivation was requested, and based on children’s responses a semantic classification was applied to distinguish between concern about global poverty and hunger, general awareness of the problem of waste, and concern about wasting their parents’ money.

The fourth phase was the collection and measurement of the leftovers of children not acting as student volunteers (n = 443). The volunteer children (n = 104) had a separate lunch prior to their peers in order to be prepared to play an active role in carrying out the analysis under the supervision of the researchers. Before the start of the lunch service, the tables and collection bins were set up by the researchers. During the lunch service, the catering staff prepared standard portions of the first course and broccoli using calibrated spoons and pre-cut cheese slices. Portion sizes did not differ among age groups. For each class, a distinct, single waste measurement session was organized. One out of four student volunteers from each class accompanied their peers to the collection table, asked them to leave their trays, and then guided them, one at a time, to another table, where a second student volunteer provided a questionnaire to collect their opinions. The other two student volunteers were responsible for separately collecting plate waste according to the categories of first course, cheese, and broccoli in specifically labeled bins. At the end of the session, the bins were weighed by the researchers using a calibrated balance in the presence of the student volunteers. The number of filled questionnaires per class was counted and it always coincided with the number of diners communicated by the teachers. No personal data were collected or used in the analysis. Waste collection was conducted anonymously, as individual plates were not measured but instead collected in a common bin, grouped by class, and subsequently weighed. The questionnaires were likewise administered anonymously, with only the class indicated on each form. No form of rating or evaluative judgment was performed.

The fifth phase consisted of processing the data collected, presenting them in the classroom and to the canteen committee, in order to discuss possible improvements.

### 2.4. Statistical Analysis

Statistical analysis was performed using Statgraphic vers. 5.1 (STCC Inc., Rockville, MD, USA). Normality assumptions were tested before applying parametric analyses. Results were reported as average value ± standard deviation (SD). To analyze the correlation between the proportion of the first course or second course and side dish wasted and the proportion of affirmative response to the question “*Yes, I wasted the first course/second course and side dish*,” a simple regression model was applied using values aggregated by age group. To compare children’s responses, ANOVA was performed on the variables amount of first course, second course and side dish wasted and responses to questions 3–6, using age group as a factor. The least significant difference (LSD, *p* < 0.05) test was subsequently applied to identify significant differences among age groups.

## 3. Results and Discussion

### 3.1. Issue 1: Were the Children Good Experimenters?

Plate waste averaged 108.4 g per child (n = 443), corresponding to 28.3% of the meal. This value is close to the average reported by the largest survey performed in Italy involving 78 primary schools, where the mean waste was 90.0 g per child per meal, corresponding to 21.7% of the food prepared every day [17]. It is particularly close to the values observed by this latter survey for the Lazio and Emilia Romagna regions, which reported 97.8 and 97.0 g of waste per child per meal, respectively [17]. This correspondence suggests that the data collected in the present study by trained young children, supervised by the researchers, are reliable, as they closely match those obtained in a large-scale survey conducted by staff personnel using a validated methodology in comparable geographic and cultural contexts [17]. Changes in the geographical location and dietary habits in which a primary school is located can lead to substantial differences in plate-waste levels. For example, a Spanish survey reported a lower average waste equal to 44 g per child per meal corresponding to 10% of meal served [18] and a Swedish survey reported 50 g per child per meal [19]. Conversely, in a Portuguese survey food waste by children at elementary school canteen was found to be 43.6% [7].

Considering the total amount of food wasted, younger students (aged 6–7 years) consumed less food than older students (aged 10–11 years) (*p* < 0.05), with the waste changing from 128.7 to 81 g per child per meal (Table 3). This age-related difference is in agreement with a previous study conducted among children of the same age range [20], but the trend is reversed for older children (aged 14 years or more) [19]. The difference in the amount of wasted meal could be due to inadequate portion sizes, which were not adjusted according to children’s ages. Moreover, food neophobia, which is the reluctance to eat or the avoidance of novel foods [21], could explain this age-related difference. Indeed, a large-scale study on the evolution of food neophobia across the lifespan showed that it peaks at approximately 5–6 years of age [22]. This trend warrants further investigation to inform appropriate interventions. Nevertheless, when differences were analyzed at the level of individual portions, age-related differences were not statistically significant (*p* > 0.05).

The waste of the main course ranged from 25.2 g per meal, representing the average value among older children, to 72.2 g per meal, representing the average value among younger children. Globally, the average waste for the first course was 23.2% of the portion served. For cheese, waste ranged from 19.8 to 27.3 g per meal (globally, 48.8%) (Table 3). Waste of vegetables fell between 29.1 and 34.1 g per meal (globally, 31.7%). The high percentage of wasted vegetables and cheese with respect to starch-based food was already observed in Italian schools where food-waste assessment was carried out by reference nutritionists in collaboration with the school canteen staff [23]. Indeed, food-waste levels were recorded at 24.4% for the first course, 31.4% for vegetable-based side dishes, 30% for grilled/primo sale cheese and 45% for stracchino/caciotta cheese [23]. These results confirm the reliability of data of the current study obtained directly by children supervised by the researchers. Moreover, they evidence that children do not follow the Mediterranean diet, which is offered in Italian schools, as already observed [17,23].

### 3.2. Issue 2: Was There a General Awareness of the Extent of Food Waste Among Children?

**Figure 2:**
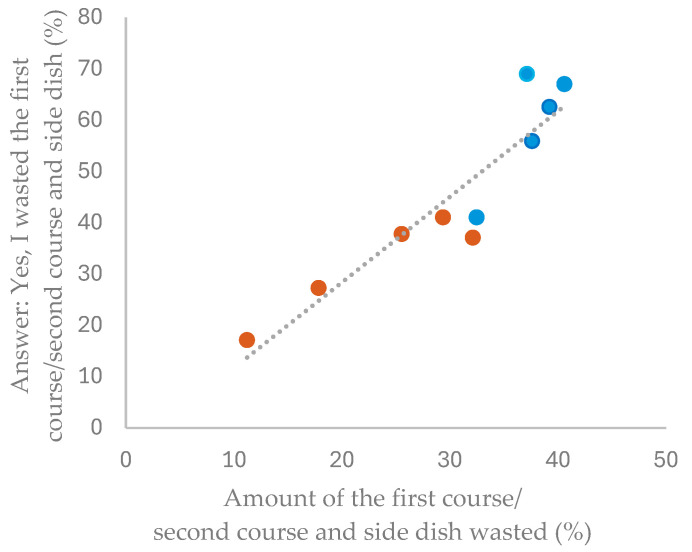
Relationship between self-reported waste of the first course (“*Yes, I wasted the first course/second course and side dish*”) and the measured percentage of first-course/second course and side dish waste based on leftover weight. Orange points (●) represent first-course waste and blue points (●) represent second-course and side-dish waste. Values are aggregated by age groups. The dotted line represents the linear relationship between measured and self-reported waste (r = 0.92, *p* < 0.001).

One aim of this study was to establish whether children were aware of their food waste. Waste collection and questionnaire administration were conducted without identifying individual children; instead, data were recorded at the class level. The relationship between plate waste and questionnaire responses was examined by comparing the total weight of plate waste with the proportion of responses, both aggregated according to children’s age groups. Using this approach, a correlation emerged between the waste of the first course and second course and side dish and the affirmative response to the questions 1: “*Have you wasted the first course/second course and side dish?*” (r = 0.92, *p* < 0.001) (Figure 2). This association should be interpreted with caution, as it does not provide insight into individual-level awareness. However, it may indicate a general tendency for responses across age groups to be broadly consistent with observed waste patterns.

### 3.3. Issue 3: Were the Children Able to Motivate the Reason for Leaving Food Uneaten?

Based on children’s responses, the motivations for leaving food uneaten were grouped according to two semantic criteria, namely “sensory dislike” and “satiety perception”; however, some responses were classified as “generic reasons.” Among the children who reported leaving food uneaten, the majority were able to explain their reasons for wasting food. In general, sensory properties played a prominent role in their decisions to leave food uneaten. Regarding the first course, specific reasons for sensory dislike accounted for 41% of the motivations, included “*It had no taste*”, “*It was cold*”, “*It was too salty*”, “*It smelled bad*”, and “*It was hard*”. With respect to the second course, specific motivations for sensory dislike accounted for 54% of the motivations and included “*I did not like broccoli*”, “*It had no taste*”, “*It was cold*”, “*It smelled bad*”, “*It was too salty*” and “*It was too greasy*”. Accordingly, in a previous study the sensory characteristics of the meal were found to be the main reason for food waste in the primary school canteen [13,24]. Excessive portion size (“*I was full*”; “*I had eaten enough*”) accounted for 33% of the motivations for wasting the first course and 11% for the second course plus side dish. Inadequate portion size is another recognized reason for food waste [25]. Generic reasons for wasting food (“*I did not like it*”; “*I didn’t feel like eating it*”) accounted for 26% in the case of the first course and 35% in the case of the second course. Food neophobia among schoolchildren likely played a role in food waste, as those who are reluctant to try unfamiliar foods generally show lower liking for school meals [26]. These latter children would benefit from familiarization actions to accept school meals. Accordingly, most children reported that they would eat the first course if it were prepared by their mother or grandmother; however, this was not the case for the second course (Table 4). Taken together, these results suggest that most children’s complaints are sufficiently clear and could be systematically collected and translated into concrete improvements, such as enhanced seasoning, better temperature control of meals, and more flexible portion sizes. However, a proportion of children still require guidance to express specific opinions about food preferences. Moreover, to address food neophobia, the development of familiarity strategies aimed at aligning school meals with those consumed at home would be necessary.

### 3.4. Issue 4: Were Children Concerned About the Impact of Food Waste?

Considering all children, on average, approximately 21.5% reported not feeling sorry when they left food uneaten, while 78.5% stated that they did feel sorry. Interestingly, among the 78.5% of children who expressed concern about food waste, 75.5% were able to explain their reasons while 3% did not provide any. Children’s motivations were grouped into two specific and one broader semantic fields based on the recurrence of the words “hunger/poverty,” “money,” or “waste” (without further specification). The primary reason (average 32.6% of total answers) was concern about world hunger (e.g., “*Yes, because there are children starving*”; “*I am sorry for poor people*”; “*I feel sorry for those who don’t have enough food*”); the second reason (28.3% of total answers) was general awareness of the problem of waste (e.g., “*It’s a waste*”, “*Food is wasted*”, “*This is going to waste*”); and the third reason (14.6% of total answers) was concern about wasting their parents’ money (e.g., “*Yes, because parents waste money*”; “*Yes, because mom and dad pay for the service*”; “*Yes, because it’s a waste of parents’ money*”) (Table 5). There was an age-related difference only in concerns about wasting money, with younger children being more concerned about this issue. The average percentage of children who would bring uneaten food home was 46.2%. Conversely, some children reported being unsure about taking leftover food home, possibly reflecting an awareness that this may not be the most effective or only solution to food waste. Approximately 27.1% of children stated that they would not bring uneaten food home.

Although the school environment is widely considered an ideal setting for hands-on nutrition education [27], the assessment of knowledge and awareness about food waste, or more generally sustainability, among young children still lacks standardized procedures. For students aged 16–22 years, the principal factors guiding food choices have been identified as sensory and emotional aspects such as mood, followed closely by price and, with some distance, health criteria, while sustainability criteria rank lowest [28]. This finding indicates that sustainability-related aspects need to be prioritized in children’s education. A few studies investigated knowledge about sustainability among primary school children. In a pilot study involving 105 students aged 9–12 years from a suburban school in Australia, a questionnaire revealed a 70% rate of correct responses about environmental questions, suggesting a need for further engagement [29]. Another study involved children aged 10–12 years in a U.S. school, who were taught six food-waste-focused lessons, resulting in significant improvements in knowledge, attitudes, self-efficacy, and behaviors among children in the intervention group compared with controls [30]. A lower target age down to 5 years was considered for an educational event in a food festival context, where pictorial responses to the prompt “*What is one action you will take to improve our food system?*” were analyzed before and after participation in educational events. Notably, limited knowledge was observed prior to the intervention, whereas after the intervention, food waste was perceived as the main issue [31]. The lack of age-related differences within the target age range of the present study indicates an absence of progressive learning on this topic and suggests that educational interventions should be effectively implemented starting from the earliest school grades.

### 3.5. Practical Implications, Limitations, and Future Research

This study adopted a child-based approach to investigate food waste. It departs from the conventional perspective that considers artificial intelligence-based procedures to be the most reliable and efficient approach for collecting large, unbiased food-waste datasets. Instead, in this study a low-cost, manual, child-based approach was adopted.

One limitation of the study lies in its exploratory nature, which does not allow for broad generalization of the findings. Consequently, the results cannot be directly extended to other geographic or socio-economic contexts.

Additional limitations are intrinsically linked to the child-centered methodology adopted. First, the selection of a specific meal—designed to ensure participation of all schoolchildren, including those with allergies and those following a vegetarian diet—may have influenced the observed waste patterns. Moreover, the aim of involving all children precluded the establishment of a control group, which could have served as a baseline to assess whether the child-driven approach effectively increased awareness of food waste.

With regard to the questionnaire, the possibility of social desirability bias cannot be excluded, as children may have reported higher levels of environmental awareness due to being observed (in the context of educational activity). Finally, the absence of a longitudinal follow-up prevented the investigation of the potential lasting effects of the child-based experimentation on children’s responses.

Nevertheless, the study yielded quantification of food waste consistent with those obtained in large-scale surveys in similar geographical and cultural contexts. Overall, teachers and canteen committee members reported being satisfied with the information provided by the study. Moreover, they had never previously quantified plate waste and were surprised by its magnitude.

Among children who reported leaving food uneaten, only a proportion (74% for the first course and 65% for the second course) were able to provide detailed reasons for disliking specific foods. Educating children to give appropriate and constructive feedback would be advisable in order to support the design of well-accepted school menus. Approximately 78.5% of students reported being aware of the implications of food waste, while the remaining participants expressed little or no concern. In this context, the canteen committee proposed strategies to further engage pupils in addressing food waste based on the principle that sustainability and health-related concepts are best learned in real-world contexts, such as during school meals.

These strategies included periodic school activities aimed at raising children’s awareness of their responsibility in food-waste generation and their potential role as drivers of change, as well as transforming the meal environment into a quieter, more dialogic, and more reflective setting. To this end, the use of circular tables for each class was proposed, with the teacher sitting among the children. This arrangement could facilitate better interaction among students, enhance supervision, and encourage discussion about the reasons for food waste. Additionally, soundproofing the dining room was suggested as a way to reduce noise, prevent shouting, and limit distractions.

## 4. Conclusions

One of the main findings of this study is that children were able to engage in food-waste assessment, as the data we collected were consistent with those obtained through validated procedures in large-scale surveys. Most children were also able to explain the reasons for their low liking of certain foods or their perception of satiety. However, a proportion of children still require guidance to express specific opinions about food preferences. Another key result is that awareness of food waste’s implications was clearly present among most of the young children, who recognized its direct effects (wasting their parents’ money), indirect effects (waste in a broader sense), and social effects (world hunger). Therefore, actions aimed at reducing food waste, even at the primary school level, should consider children as “active” subjects who evaluate the context and collaborate in identifying strategies to reduce waste. There is a need, in future research, to define a baseline for children’s awareness and concern about their own food waste, which could serve as an indicator of both the urgency and the effectiveness of educational interventions. Finally, considering the dynamic nature of learning, school meals should be framed as systematic educational moments rather than merely rest activities.

## Figures and Tables

**Table 1:** Quiz game proposed to the students in the third, fourth, and fifth grades of the primary school.

1. *Adele only has eggs, spinach, and cheese in the fridge.* *I don’t know what to cook with these ingredients. What would you suggest?* A. Going grocery shopping. B. A cake. C. An omelette.
2. *For dinner, the mother prepared too much meat ragù. What should she do?* A. Throw it away. B. Give it to the dog. C. Put it in the fridge for the next day.
3. *If you have leftover snacks at school, what do you do?* A. I throw it in the bin. B. I take it home. C. I offer it to a classmate.
4. *If your mother cooks lasagna, what do you do?* A. I take only one slice. B. I take a very large slice. C. I take one slice and ask for seconds.
5. *If you see a dish for the first time, what do you do?* A. I taste it. B. I leave it without even tasting it. C. I let someone else taste it first.
6. *How much do you think the food thrown away in your school canteen in one day weighs?* A. Like a dad B. Like a car C. Like two backpacks full of books.

**Table 2:** Questionnaire administered to 443 schoolchildren aged 6–11 years.

**Items: Children’s Awareness of the Extent of Their Own Food Waste and Children’s Ability to Motivate the Reasons for Leaving Food Uneaten**	
Questions	Answers’ classification
*1.* *Have you wasted the first course?* *If yes: Why? (Provide a single reason only)*	Yes/No If yes, motivations were grouped according to semantic criteria
*2.* *Have you wasted the second course and side dish?* *If yes: Why? (Provide a single reason only)*	Yes/No If yes, motivations were grouped according to semantic criteria
*3.* *If the same main course is cooked by your mother or grandmother,* *do you eat it?*	Yes/No/Not sure
*4.* *If the same second course is cooked by your mother or grandmother,* *do you eat it?*	Yes/No/Not sure
**Item: Children’s Concern About the Impacts of Food Waste**	
Questions	Answers’ classification
*5.* *When it happens that you leave some food uneaten, are you sorry?* *If yes: Why? (Provide a single reason only)*	Yes/No If yes, motivations were grouped according to semantic criteria
*6.* *Would you like to take home what you leave?*	Yes/No/Not sure

**Table 3:** Food waste recorded directly by children under researcher supervision at two elementary schools ^1^.

		First Course (Rice)	Second Course (Cheese)	Side Dish (Broccoli)	Total
Age	n	g/Child (% Portion)	g/Child (% Portion)	g/Child (% Portion)	g/Child (% Meal)
6–7	64	72.2 ^a^ ± 4.3 (32.1 ± 1.9)	27.3 ^a^ ± 6.0 (54.7 ± 12.0)	29.1 ^a^ ± 7.7 (29.1 ± 7.7)	128.7 ^a^ ± 2.5 (34.3 ± 0.7)
7–8	78	40.2 ^a^ ± 11.6 (17.8 ± 5.2)	25.6 ^a^ ± 8.9 (51.3 ± 17.8)	33.2 ^a^ ± 7.8 (33.2 ± 7.8)	99.0 ^ab^ ± 10.6 (26.4 ± 2.8)
8–9	80	57.4 ^a^ ± 3.6 (25.5 ± 1.6)	26.8 ^a^ ± 1.5 (53.6 ± 3.1)	34.1 ^a^ ± 12.4 (34.1 ± 12.4)	118.3 ^ab^ ± 14.5 (31.6 ± 3.9)
9–10	101	66.1 ^a^ ± 17.8 (29.4 ± 7.9)	19.8 ^a^ ± 14.4 (39.7 ± 28.5)	28.9 ^a^ ± 1.9 (28.9 ± 1.9)	114.8 ^ab^ ± 30.1 (30.6 ± 8.0)
10–11	120	25.2 ^a^ ± 26.7 (11.2 ± 11.9)	22.4 ^a^ ± 21.3 (44.9 ± 42.6)	33.3 ^a^ ± 9.0 (33.3 ± 9.0)	81.0 ^b^ ± 14.4 (21.6 ± 3.8)
All	443	52.2 ± 21.6 (23.2 ± 5.7)	24.4 ± 9.8 (48.8 ± 15.3)	31.7 ± 6.7 (31.7 ± 6.7)	108.4 ± 21.7 (28.3 ± 5.8)

^1^ Values are expressed as mean ± SD within each age group for the amount of portion wasted and the corresponding percentage. n indicates the number of participating children in each age group. Portion waste percentages were calculated by taking the weight of standard portions provided to students by the catering staff, using calibrated utensils (for first courses and side dishes) or pre-cut slices (for cheese) as 100%. Different superscript letters within the same column indicate statistically significant differences among age groups according to Fisher’s least significant difference (LSD) post hoc test (*p* < 0.05).

**Table 4:** Children’s answers to questions 3 and 4 ^1^.

		*3. If the Same Main Course Is Cooked by Your Mother or Grandmother, Do You Eat It?*			*4. If the Same Second Course Is Cooked by Your Mother or Grandmother, Do You Eat It?*		
Age	n	Yes (%)	Not Sure (%)	No (%)	Yes (%)	Not Sure (%)	No (%)
6–7	64	81.8 ± 4.0	15. 0 ± 4.9	3.2 ± 0.9	66.9 ± 2.1	20.1 ± 1.3	13.0 ± 3.4
7–8	78	67.5 ± 32.3	14.9 ± 12.0	17.6 ± 20.3	53.6 ± 24.6	28.3 ± 8.1	18.1 ± 16.5
8–9	80	61.0 ± 10.8	26.2 ± 0.7	12.9 ± 10.1	37.0 ± 8.3	37.5 ± 0.4	25.6 ± 7.9
9–10	101	73.1 ± 1.4	22.0 ± 1.8	4.9 ± 0.4	40.5 ± 1.3	30.6 ± 0.6	28.9 ± 1.9
10–11	120	78. 8 ± 5.4	15.8 ± 0.6	5.4 ± 0.4	39.8 ± 29.0	36.4 ± 32.8	23.8 ± 3.8
All	443	72.4 ± 14.0	18.8 ± 6.5	8.8 ± 9.7	47.5 ± 17.6	30.6 ± 13.1	21.9 ± 8.7

^1^ Values are expressed as mean ± SD within each age group. n indicates the number of participating children in each age group. For both questions 3 and 4, percentages indicate the proportion of children selecting each response option. No statistically significant differences among age groups were observed according to Fisher’s least significant difference (LSD) post hoc test (*p* < 0.05).

**Table 5:** Children’s answers to questions 5 and 6 ^1^.

		*5. When It Happens That You Leave Some Food Uneaten,* *Are You Sorry? If Yes, Why? (Provide a Single Reason Only)*				*6. Would You Like to Take Home* *What You Leave?*		
Age	n		Yes (%)		No (%)	Yes (%)	Not Sure (%)	No (%)
		Hunger/Poverty	Waste	Money				
6–7	64	15.4 ^a^ ± 1		31 ^a^ ± 3	35.0 ^a^ ± 8	46.6 ^a^ ± 21.2	20.9 ^a^ ± 7.7	32.6 ^a^ ± 13.5
7–8	78	27.8 ^a^ ± 6.3	24.0 ^a^ ± 11.2	23.0 ^ab^ ± 0.6	17.1 ^a^ ± 5.9	53.5 ^a^ ± 15.5	24.1 ^a^ ± 7.0	22.4 ^a^ ± 22.5
8–9	80	21.7 ^a^ ± 5.6	41.9 ^a^ ± 6.7	14.4 ^bc^ ± 7.9	21.9 ^a^ ± 6.7	49.0 ^a^ ± 3.4	29.7 ^a^ ± 10.6	21.3 ^a^ ± 13.9
9–10	101	47.5 ^a^ ± 28.2	31.5 ^a^ ± 11.6	5.5 ^c^ ± 4.5	13.0 ^a^ ± 8.5	43.3 ^a^ ± 2.1	32.7 ^a^ ± 19.5	23.9 ^a^ ± 17.4
10–11	120	41.8 ^a^ ± 27.9	20.1 ^a^ ± 1.4	7.3 ^c^ ± 2.2	27.4 ^a^ ± 27.8	38.7 ^a^ ± 13.2	25.9 ^a^ ± 9.6	35.4 ^a^ ± 22.8
All	443	32.6 ± 18.8	28.3 ± 10.9	14.6 ± 9.8	21.5 ± 13.0	46.2 ± 11.2	26.7 ± 9.8	27.1 ± 15.0

^1^ Values are expressed as mean ± SD within each age group. n indicates the number of participating children in each age group. For question 5, the reasons provided by children were grouped according to semantic criteria, and percentages under each reason category were calculated over the total number of children in the age group. Some of these participants left the reason blank; therefore, the percentages of ‘Yes’ and ‘No’ do not sum to 100%. For question 6, percentages indicate the proportion of children selecting each response option. Different superscript letters within the same column indicate statistically significant differences among age groups according to Fisher’s least significant difference (LSD) post hoc test (*p* < 0.05).

## Data Availability

The original contributions presented in this study are included in the article. Further inquiries can be directed to the corresponding author.

## References

[1] Nocentini E., Marchi M., Caro D., Tropea F., Patrizi N., Pulselli F.M. (2026). Calculation of Life Cycle-Based Emission Factors for Administrative Services: A Replicable Framework for Climate Action in the Public Sector. Environ. Impact Assess. Rev..

[2] Tregear A., Aničić Z., Arfini F., Biasini B., Bituh M., Bojović R., Brečić R., Brennan M., Barić I.C., Del Rio D. (2022). Routes to Sustainability in Public Food Procurement: An Investigation of Different Models in Primary School Catering. J. Clean. Prod..

[3] Eustachio Colombo P., Patterson E., Lindroos A.K., Parlesak A., Elinder L.S. (2020). Sustainable and Acceptable School Meals through Optimization Analysis: An Intervention Study. Nutr. J..

[4] Gutkowska K., Czarniecka-Skubina E., Górska-Warsewicz H., Hamulka J. (2025). Nutrition Knowledge of Primary Schoolchildren in Poland from the Parents’ perspective Based on Qualitative Studies. Sci. Rep..

[5] Yeo G.S., Lee S.T., Wong J.E., Khouw I., Safii N.S., Poh B.K. (2024). SEANUTS II Malaysia Study Group. Association of Breakfast Skipping on Nutrient Intake and Adiposity in Malaysian Children: Findings from SEANUTS II. Appetite.

[6] Danielzik S., Pust S., Landsberg B., Muller M.J. (2005). First Lessons from the Kiel Obesity Prevention Study (KOPS). Int. J. Obes..

[7] Abreu M., Carvalho J., Gonçalves C. (2025). Evaluation of Nutritional and Economic Impact of Food Waste in a School Canteen. Meas. Food.

[8] Petchoo J., Kaewchutima N., Tangsuphoom N. (2022). Nutritional Quality of Lunch Meals and Plate Waste in School Lunch Programme in Southern Thailand. J. Nutr. Sci..

[9] Sundin N., Halvarsson R., Scherhaufer S., Schneider F., Eriksson M. (2024). From Plate to Waste: Composition of School Meal Waste and Associated Carbon Footprint and Nutrient Loss. Resour. Conserv. Recycl..

[10] Patra E., Kokkinopoulou A., Pagkalos I. (2023). Focus of Sustainable Healthy Diets Interventions in Primary School-Aged Children: A Systematic Review. Nutrients.

[11] Calì R., Ferreira J., Cerqueira P., Ribeiro J., Valente de Oliveira J., Leite J., Rodrigues J., Dias J., Cardoso P. (2026). Food Waste Detection in Canteen Plates with Visual Large Language Models. Progress in Artificial Intelligence.

[12] Poelman A.A.M., Djakovic S., Heffernan J.E., Cochet-Broch M., Golley R.K., Cox D.N., Beelen J. (2022). Effectiveness of a Multi-Strategy Behavioral Intervention to Increase Vegetable Sales in Primary School Canteens: A Randomized Controlled Trial. Nutrients.

[13] Martins L.M., Rodrigues S.S., Cunha L.M., Rocha A. (2021). School Lunch Nutritional Adequacy: What is Served, Consumed and Wasted. Public Health Nutr..

[14] García-Herrero L., De Menna F., Vittuari M. (2019). Food Waste at School. The Environmental and Cost Impact of a Canteen Meal. Waste Manag..

[15] Antón-Peset A., Fernandez-Zamudio M.-A., Pina T. (2021). Promoting Food Waste Reduction at Primary Schools. A Case Study. Sustainability.

[16] Sundin N., Malefors C., Strotmann C., Orth D., Kaltenbrunner K., Obersteiner G., Scherhaufer S., Sjölund A., Persson Osowski C., Strid I. (2024). Sustainability Assessment of Educational Approaches as Food Waste Prevention Measures in School Catering. J. Clean. Prod..

[17] Falasconi L., Boschini M., Giordano C., Cicatiello C., Alboni F., Nassivera F., Troiano S., Marangon F., Segrè A., Franco S. (2025). Who Cleans the Plate? Quantity and Type of Food Waste in 78 Primary Schools’ Canteens in Italy. Sustainability.

[18] Vidal-Mones B., Diaz-Ruiz R., Gil J.M. (2022). From Evaluation to Action: Testing Nudging Strategies to Prevent Food Waste in School Canteens. Waste Manag..

[19] Malefors C., Gerstbrein T., von Brömssen C., Sundin N., Ran Y., Lambe F., Eriksson M. (2026). Understanding Food Waste Generation in School Canteens. Environ. Dev..

[20] Niaki S.F., Moore C.E., Chen T.A., Cullen K.W. (2017). Younger Elementary Students Waste more School Lunch Foods than Older Elementary Students. J. Acad. Nutr. Diet..

[21] Pliner P., Hobden K. (1992). Development of a Scale to Measure the Trait of Food Neophobia in Humans. Appetite.

[22] Hazley D., Stack M., Walton J., Mcnulty B.A., Kearney J.M. (2022). Food Neophobia across the Life Course: Pooling Data from Five National Cross-Sectional Surveys in Ireland. Appetite.

[23] Vici G., Giustozzi D., Camilletti D., Zufolino S., Malandrino L., Renzi S., Pucciarelli S., Vincenzetti S., Belli L., Polzonetti V. (2025). An Evaluation and Optimization of Nutrition, Environmental Footprint, and Food Waste in Italian Primary School Menus: A Case Study. J. Transl. Med..

[24] Martins L.M., Rodrigues S.S.P., Cunha L.M., Rocha A. (2020). Factors Influencing Food Waste during Lunch of Fourth-grade School Children. Waste Manag..

[25] Boschini M., Falasconi L., Cicatiello C., Franco S. (2020). Why the Waste? A Large-Scale Study on the Causes of Food Waste at School Canteens. J. Clean. Prod..

[26] Piochi M., Fino M.A., Torri L. (2025). The Impact of Children’s Food Neophobia on Meal Perception, Emotional Responses, and Food Waste in Italian Primary School Canteens. Foods.

[27] Czarniecka-Skubina E., Hamulka J., Jeruszka-Bielak M., Gutkowska K. (2024). Do Food and Meal Organization Systems in Polish Primary Schools Reflect Students’ Preferences and Healthy and Sustainable Dietary Guidelines? The Results of Qualitative Research for the Junior-Edu-Żywienie (JEŻ) Project. Foods.

[28] Zorell C.V. (2022). Central Persons in Sustainable (Food) Consumption. Int. J. Environ. Res. Public Health.

[29] Muldoon R., Shelford T., Holland O., Hryciw D.H. (2019). Environmental Awareness of Primary School Aged Children in Brisbane, Australia. Int. J. Innov. Sci. Math. Educ..

[30] Elnakib S., Subhit S., Shukaitis J., Rowe A., Cava J., Quick V. (2024). New Jersey Leaves No Bite Behind: A Climate Change and FoodWaste Curriculum Intervention for Adolescents in the United States. Int. J. Environ. Res. Public Health.

[31] Barbour L., Tan W.C., Welch R.K., Leahy D. (2026). Engaging children in food systems literacy research: An analysis of children’s drawings from the Little Food Festival. Health Educ..

